# Development of Molecularly Imprinted Photonic Crystals Sensor for High-Sensitivity, Rapid Detection of Sulfamethazine in Food Samples

**DOI:** 10.3390/polym17020160

**Published:** 2025-01-10

**Authors:** Jinxing He, Mengke Wu, Xin Wang, Ruoxuan Xu, Shuting Zhang, Xiaolei Zhao

**Affiliations:** Shandong Key Laboratory of Healthy Food Resources Exploration and Creation, School of Food Science and Engineering, Qilu University of Technology (Shandong Academy of Sciences), Jinan 250353, China

**Keywords:** photonic crystal, molecular imprinting, sulfamethazine, sensor

## Abstract

As a veterinary drug, sulfamethazine is frequently used to control animal diseases. In this study, a novel molecularly imprinted photonic crystal sensor for the fast visual detection of sulfamethazine in milk and chicken has been developed. Under optimum preparation conditions, a molecularly imprinted, photonic crystal with an anti-opal structure and a clear bright color was prepared and characterized. The adsorption conditions, including adsorption solvent, solvent pH, and detection time, were studied in detailed. Based on its excellent selectivity and fast response, a photonic crystal sensor detection method for the quantitative analysis of sulfamethazine was established, which achieved good linearity, ranging from 10^−4^ mg/L to 10 mg/L, a limit detection of 1.16 μg/L, and spiked recoveries of 80.56% to 103.59%, with a relative standard deviation (RSD) <6.41%. More importantly, the detection process could be completed within 3 min. This method provides an alternative for the rapid screening of sulfamethazine in food samples.

## 1. Introduction

Sulfonamides (SAs) are commonly used as antibiotics in the aquaculture industry. Because of the advantages of low price, stability, and broad-spectrum antimicrobial properties, they are widely used for the prevention and treatment of bacterial infectious diseases [1]. However, excessive levels of SAs in food are harmful to human health, leading to liver toxicity, urinary stones, kidney failure, or allergic reactions [2]. To confirm the food safety for consumers, the European Union and China have established the maximum residue level of total SAs in animal-derived food at 100 μg/kg, and China has also set a standard that the residue level of sulfamethazine (SMZ) as a single drug should not exceed 25 μg/kg [3].

At present, instrument-based analytical approaches, including high-performance liquid chromatography (HPLC), liquid chromatography coupled with mass spectrometry, enzyme-linked immunosorbent assay (ELISA), and spectrophotometric methods, are still the most commonly used methods for detection of SAs in food samples [2,4]. Although these methods can be performed with high accuracy, the disadvantages of complicated operations, expensive instruments and specialized personnel training still limit their application for fast and on-site detection [5]. 

Based on the semiconductor crystal and electronic bandgap, the photonic crystal technique was first presented in 1987. When different dielectric materials are periodically arranged, the electromagnetic waves can form a photonic band or stop band during the propagation process due to Bragg reflection [6]. There can be a similar band gap between photon energy bands and the photonic band gap, which only allows a certain frequency of light to propagate through the material, while other wavelength light is reflected. When loading different substances, the diffraction peaks of photonic crystals are shifted, which enables their detection. 

To date, photonic crystals mainly include opal and anti-opal photonic crystals. During the preparation of anti-opal photonic crystals, they are formed by filling the voids of and opal photonic crystal with highly refractive index materials, and then the original materials of the opal structure are removed [7]. Even though multiple additional steps (such as colloidal self-assembly, template ablation or chemical etching) are required, the anti-opal photonic crystals still exhibit outstanding advantages as follows: (1) Because of the periodic micro- and nanopores, they are able to generate structural colors by Bragg diffraction and label-free detection. (2) With the removal of the original spheres, the anti-opal photonic crystals just retain the backbone materials, which can enhance the mass transfer of analyte and reduce the response time [8,9]. Based on the advancements in anti-opal photonic crystals, their application in sensor detection has made significant progress. During the practical application of sensors, the selectivity for the target is still a difficult challenge because of the complex nature of the food matrix, yet that is the absence of photonic crystal sensors [10].

In recent decades, the molecular imprinting technique (MIT) has become a well-established technology for the preparation of novel polymeric materials with predictable structures and specific adsorption for a template [11,12]. Molecularly imprinted photonic crystals (MIPC) sensors are achieved by introducing target molecules into the gaps of the photonic crystal film and then removing the target molecules [13]. The obtained MIPC sensors combine the excellent specific adsorption from imprinted polymers and the quick response of photonic crystals. Zhang et al. combined the molecular imprinting technique, photonic crystallography, and fluorescent materials to construct a series of four-channel sensor arrays, which could rapid identify five SAs in the sample within 200 s [14]. Liu et al. developed a portable vision sensor based on photonic crystallization and molecular imprinting polymers, and the amount of butyl paraben in the toner sample could be estimated by comparing the actual color of the MIPC with a “color guide”. The content of butyl paraben could be recorded and quantified by a fiber spectrometer, and the detection limit was 0.022 mmol/L [15]. 

**Figure 1:**
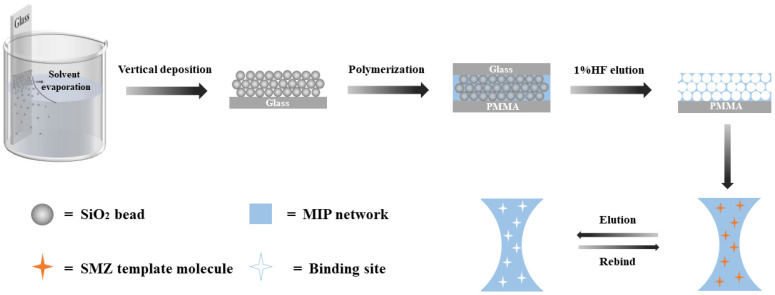
Preparation process of SMZ molecularly imprinted, photonic crystal sensor.

Based on the above considerations, the aim of this study was to develop a molecularly imprinted, photonic crystals sensor for the quantification analysis of SMZ in milk and chicken, with high selectivity, a fast response, and excellent accuracy. The characterization and adsorption performance of the MIPC, the optimum detection conditions, selectivity, method validation, and practical application in food samples were all investigated in detail. The fabrication process of the MIPC sensor is shown in Figure 1.

## 2. Materials and Methods

### 2.1. Reagents and Materials

SMZ, sulfadiazine (SD), sulfisoxazole (SIZ), and ethylene glycol dimethacrylate (EGDMA) were all purchased from Aladdin Reagent Co., Ltd. (Shanghai, China). Azobisisobutyronitrile (AIBN) and methacrylic acid (MAA) were purchased from Sinopharm Chemical Reagent Co., Ltd. (Shanghai, China). Ethanol, methanol, and acetic acid were purchased from Xilong Chemical Co., Ltd. (Shantou, China). Milk and chicken were purchased from a local supermarket in Jinan, Shandong Province. Prior to use, AIBN should be purified, as reported in the literature [16]. The other chemicals could be used directly.

### 2.2. Instruments

Reflectance spectrum measurement was performed on a fiber optic spectrometer (PG 2000, Shanghai Fuxiang Optical Co., Ltd., Shanghai, China). Dual beam ultraviolet–visible spectrophotometry (TU-1950, Beijing General Instrument Co., Ltd., Beijing, China), and scanning electron microscopy (SEM, S4800, Hitachi, Tokyo, Japan) were used to characterize the polymers. The PC growth was performed in an incubator (Yiheng Science Instrument Co., Ltd., Shanghai, China) at a constant temperature of 40 °C and constant humidity of 60%.

### 2.3. Preparation of SiO_2_ Microspheres

Monodisperse SiO_2_ microspheres with uniform particle size were synthesized using the traditional Stöber method [17]. First, 6 mL of ammonia was added to 16.25 mL of ethanol and 24.75 mL of distilled water solution. Subsequently, 4.5 mL of tetraethyl orthosilicate/anhydrous ethanol solution (*v*/*v*, 1:10) was rapidly poured into the above ammonia solution under continuous stirring and reacted for 2 h. After being washed with anhydrous ethanol for three times, the resulting particles were dried to a constant weight, resulting in monodisperse SiO_2_ microspheres.

### 2.4. Preparation of MIPC Sensor for SMZ

In order to obtain the MIPC for SMZ, a glass slide was first cleaned with piranha solution (98% H_2_SO_4_: H_2_O_2_ = 7:3, *v*/*v*) for 24 h and then washed with sufficient distilled water to neutralize the piranha solution. After that, the obtained glass slide was vertically soaked in the as-prepared SiO_2_ microspheres/anhydrous ethanol solution with a concentration of 0.3% (*w*/*w*). The mixed system was maintained at 60 °C and air humidity of 40% for 72 h.

Then, 27.8 mg of SMZ as template, 33.9 μL of MAA as functional monomer, 495 μL of EGDMA as cross-linker, and 15 mg of AIBN as initiator were added to 2 mL of methanol solution, purged for 10 min with nitrogen, and sonicated for 10 min to form the imprinted precursor solution. A polymethylmethacrylate (PMMA) plate was placed on a glass plate of PC to form a “sandwich” structure. Subsequently, a small amount of precursor solution was added dropwise into the interstitial space between the glass slide and PMMA layer until the photonic crystal layer became transparent. The polymerization reaction was induced at 60 °C for 4 h in the dark. To remove the SiO_2_ microspheres, the obtained PC plate was etched in 1% HF solution for 4 h, and the template SMZ was further eluted with acetic acid and methanol (1:9 *v*/*v*) until SMZ in the eluent could not be detected. Finally, the MIPC sensor was stored in phosphate buffer solution (0.01 mol/L, pH 6.5).

As a control, a non-imprinted polymer photonic crystal (NIPC) sensor was also prepared with the same process except the template SMZ addition. 

### 2.5. Reflection Spectrum Measurement

When the highly cross-linked polymers were dispersed into the solvent, the solvent could diffuse into the interior of polymers, and the polymers also could absorb the solvent, causing volume expansion [18]. When the concentration of solvent reached the maximum and saturated state, the swelling equilibrium was also achieved. In this study, the molecularly imprinted photonic crystal polymers are a kind of highly cross-linked polymer, and the optical properties of the MIPC (such as the diffraction peak position and photon band gap) depend on their pore structure [19,20]. Before the assay, it was necessary that the MIPC and NIPC were immersed in phosphate buffer solution at pH = 6.5 overnight to reach swelling equilibrium and avoid the impact of volume swelling of polymers on optical performance. The fiber optic probe of the spectrometer was placed above the PC surface and kept vertical. The concentration of SMZ was gradually increased from 10^−4^ mg/L to 10^3^ mg/L, and the PC reactions were recorded. All experiments were performed under the same conditions.

### 2.6. Detection of SMZ in Real Samples

#### 2.6.1. Milk Samples

Samples of 5.0 mL were added to a 10 mL centrifugal tube, and centrifuged at 8000× *g* rpm for 10 min to remove the liquid layer. After that, the remaining solution was added to 5 mL of methanol to remove the excess protein, and the supernatant was collected and dried with N_2_ to near dryness at room temperature. Finally, the remaining part was re-dissolved with 5 mL of phosphate buffer solution at pH 6.5 for analysis by reflectance spectroscopy.

#### 2.6.2. Chicken Samples

For the chicken samples, 5.0 g was accurately weighed, and then, 1.5 mL of hydroxylamine hydrochloride and 3.5 mL of ammonium acetate (50 mmol/L, pH = 4.5) were added to the chicken sample. After being shaken for 5 min at 8000 rpm, 2 mL of acetonitrile solution was further added. The mixture was then sonicated for 5 min and centrifuged at 10,000× *g* rpm for 5 min [17]. Subsequently, the supernatant was collected and dried with N_2_ to near dryness at room temperature. The remaining part was re-dissolved with 5 mL of phosphate buffer solution at pH 6.5 for analysis by reflectance spectroscopy. During the detection process, the same batch of fresh chicken samples were selected and weighed for each experiment.

In addition, a spiked recovery experiment with SMZ solution at four levels (1, 10, 10^2^, 10^3^ µg/L) was also conducted. Meanwhile, the intra-day and inter-day experiments were all conducted, in which the intra-day experiment was performed by analyzing the spiked samples prepared within a day, whereas the inter-day experiment assessed the spiked samples prepared on three different days.

## 3. Results and Discussion

### 3.1. Optimization of Preparation Conditions for MIPC

**Figure 2:**
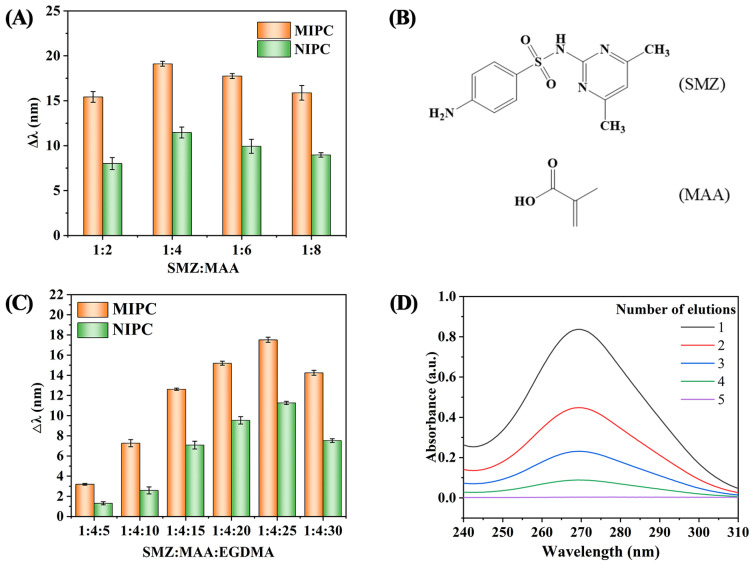
The reflectance peak change (Δλ) value of MIPC/NIPC at different ratios of (**A**) SMZ/MAA and (**C**) SMZ/MAA/EGDMA. (**B**) Chemical structure of SMZ and MAA. (**D**) The absorption spectroscopy of SMZ under several adsorption–desorption elutions.

The ratio of template molecules to functional monomers is the most important factor affecting the formation of efficiently imprinted sites. In this study, the effect of different ratios of SMZ and MAA (1:2, 1:4, 1:6, and 1:8) on their changes in reflected wavelengths (Δλ) in 10^2^ μg/L SMZ solution were evaluated, as shown in Figure 2A. The largest change in reflected wavelengths occurred at a 1:4 SMZ/MAA ratio. Based on the structure of SMZ and MAA (see Figure 2B), the hydrogen bond interaction between the amino group from SMZ and the carboxyl group from MAA is the main force in achieving the imprinted sites [21]. At a low ratio, a low template to functional monomer mixture with fewer imprinted sites was formed, while ratios of 1:6 and 1:8 produced a high number of non-specific adsorption sites [22]. In the subsequent experiment, a 1:4 ratio of SMZ/MAA was selected to prepare the SMZ imprinted layer.

Except for the SMZ/MAA ratio, the amount of cross-linker agent is another important factor contributing to the toughness of the MIPs and MIPC sensor. In this study, EGDMA was selected as the cross-linker. Under a constant SMZ/MAA ratio, different amounts of EGDMA (SMZ: MAA: EGDMA, 1:4:5, 1:4:10, 1:4:15, 1:4:20, 1:4:25, and 1:4:30) were used to prepare different imprinted layers, and the reflected wavelengths changes (Δλ) were recorded on the fiber-optic spectrometer. From Figure 2C, we can see that the maximum shift of 17.5 nm was observed at the SMZ/MAA/EGDMA ratio 1:4:25. When the ratio changed to 1:4:30, the Δλ value was decreased. In the preparation of imprinted polymers, increased amounts of cross-linker could result in excessive cross-linker domains, leading to a slow optical response [23]. Therefore, the molar ratio of 1:4:25 was selected as the optimum conditions of SMZ/MAA/EGDMA to prepare the imprinted layer in the subsequent experiments.

In addition, the efficiency elution of the target molecule SMZ is another key factor. To confirm the elution conditions, the as-prepared MIPC sensor was soaked in acetic acid/methanol solution (1:9, *v*/*v*) and placed on a shaker at 100 rpm to remove the SMZ. During the elution, the eluent solution was frequently changed (every 2 min) and measured with a UV spectrophotometer to monitor the SMZ absorption spectroscopy in the elution. As shown in Figure 2D, the absorption peaks of SMZ gradually decreased as the number of elutions increased, and the UV absorption peaks of SMZ disappeared after five elutions, indicating that the template molecule SMZ was completely removed.

### 3.2. Characterization of the MIPC Sensor

#### 3.2.1. Morphology Characterization of SiO_2_ Microspheres and the MIPC Sensor

**Figure 3:**
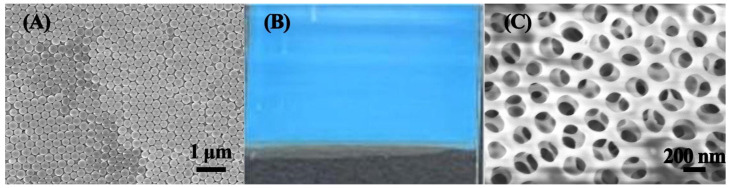
(**A**) SEM image of SiO_2_ photonic crystal film. (**B**) Digital image of PC film. (**C**) SEM image of MIPC.

During the construction of the MIPC sensor, the anti-opal structure was formed by HF etching of the SiO_2_ microspheres. SiO_2_ microspheres were first synthesized by the Stöber method. As the SEM image shows in Figure 3A, the SiO_2_ microspheres exhibited a uniform sphere structure and were regularly arranged to form an ordered structure. Meanwhile, the obtained PC film showed a clear bright blue color (see Figure 3B). Subsequently, the SiO_2_ microspheres were vertically deposited to fabricate photonic crystal films featuring vivid structural color. The molecularly imprinted precursor solution penetrated into the void between the PC and the PMMA plate by capillary force. After molecularly imprinted polymers were generated, 1% HF solution was used to remove the SiO_2_ framework to form an anti-opal structure, which is also shown in Figure 3C. The unique anti-opal structure could promote a faster mass transfer rate and shorter detection times. The above results all indicate the successful construction of the MIPC sensor. 

#### 3.2.2. Reflection Spectra Characterization

**Figure 4:**
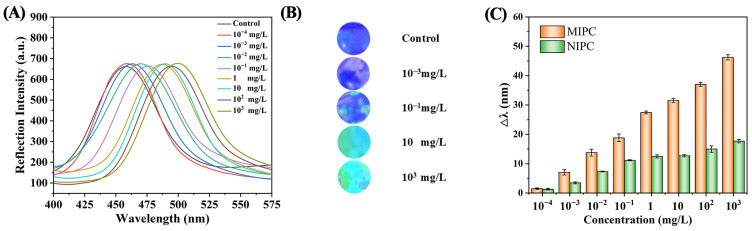
(**A**) The reflectance peak of MIPC under different concentrations of SMZ ranging from 10^−4^ mg/L to 10^3^ mg/L. (**B**) The structural color of MIPC after loading SMZ solution. (**C**) The reflectance peak change (Δλ) value of MIPC/NIPC under different concentrations of SMZ.

A fiber-optic spectrometer was used to detect the changes in the diffraction peak wavelengths of the MIPC in different concentrations of SMZ, as shown in Figure 4A. When immersed in blank buffer solution, the maximum absorption peak of the MIPC sensor was 457 nm. When the concentration of SMZ increased from 10^−4^ mg/L to 10^3^ mg/L, the diffraction peak wavelength gradually red-shifted, and the maximum Δλ value was 46 nm. Based on Bragg’s law, the maximum diffraction wavelength λ_max_ follows Equation (1).

(1) $$\lambda_{max}=1.633\left(d/m\right){\left(n_{a}^{2}-\sin^{2}\theta \right)}^{1∕2}$$

where *n_a_* means the average refractive index of the material the MIPC is composed of, *d* is the crystal plane spacing, and *m* is the Bragg diffraction order. When the target molecule is bonded in the imprinted sites, both *n_a_* and *d* change and then cause a wavelength shift [24]. 

Meanwhile, due to the λ_max_ red-shift, the structural color of the MIPC under different concentrations of SMZ also changes from blue to bluish-green and then gradually becomes green, suggesting that the visual detection of SMZ could be easily achieved with the MIPC sensor through the conversion of the concentration parameter into optical signals (see Figure 4B).

To confirm the existence of imprinted sites, the NIPC sensor was also tested under the same conditions as the MIPC sensor. As shown in Figure 4C, similar trends for the NIPC/MIPC sensors were observed, with wavelength shifts gradually increasing with the increasing SMZ concentrations. The NIPC exhibited a maximum shift of 17.0 nm, which is much smaller than that of the MIPC sensor, indicating that the adsorption ability of MIPC to SMZ was clearly higher. This may result from the amount of specific recognition sites in MIPC. During the preparation of imprinted polymers, the obtained imprinted sites are highly compatible with the molecular shape, size, and chemical properties of SMZ, while the NIPC sensor mainly depends on non-specific adsorption.

### 3.3. Optimization of Adsorption Conditions for MIPC

#### 3.3.1. Effect of Adsorption Solvent

Considering that methanol was used as a porogen during the preparation of the molecularly imprinted layer, it was also used as the adsorption solvent to reduce the swelling effect of the MIPC sensor. 

**Figure 5:**
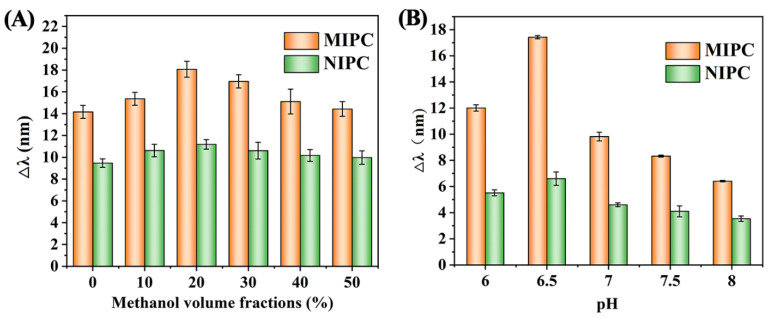
The reflectance peak change (Δλ) value of MIPC/NIPC at (**A**) different solvents, and (**B**) different solvent pH.

The sensor was fully immersed in 10^2^ μg/L SMZ solution containing 0%, 10%, 20%, 30%, 40%, and 50% methanol in phosphate buffer for 3 min, and the optimal solvent conditions were determined by observing the reflected wavelength change. As shown in Figure 5A, the Δλ value of the sensor reached a maximum of 11.2 nm at 20% methanol solution, indicating that the sensor had the maximum signal response and the best detection performance for SMZ under this condition. Therefore, 20% methanol/phosphate buffer solution was selected as the adsorption solvent.

#### 3.3.2. Effect of Solvent pH

The pH of the solution system is another factor affecting the re-adsorption process, and the SMZ standard solution at a concentration of 10^2^ μg/L was chosen as the working solution to optimize the effect of pH. The results are shown in Figure 5B. The Δλ value reached a maximum of 17.4 nm at pH 6.5 and gradually decreased at pH above 6.5. These results gave direct evidence that the maximum adsorption capacity of the MIPC film on SMZ was found. From the structure of SMZ, as shown in Figure 2B, we can see that the analyte SMZ consists of two ionizable functional groups, including an aromatic amine and a sulfonamide group, with two pKa values (pKa_1_ = 2.31; pKa_2_ = 7.58) [25]. Under weak acidic conditions, the SMZ are in a molecular state, which could increase the adsorption performance of SMZ, yet the ionic state of SMZ at pH above 6.5 could reduce the adsorption capacity [26,27]. Consequently, pH 6.5 was selected as the adsorption solvent pH for the further study.

### 3.4. Performance Analysis of MIPC Sensor

#### 3.4.1. Optimum Detection Time

To ensure the complete adsorption between imprinted sites and target SMZ, the MIPC and NIPC sensors were immersed into SMZ solution at a concentration of 10^2^ μg/L. The reflected wavelengths were detected every 30 s, and the effect of contact time on diffraction wavelength was studied from 0 to 5 min. 

**Figure 6:**
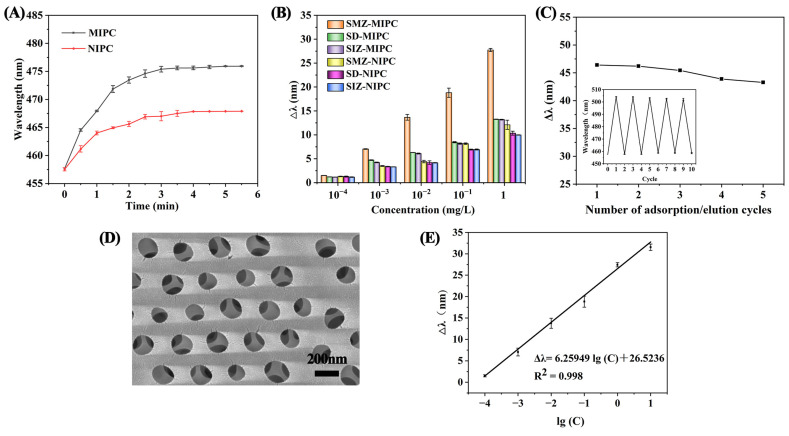
(**A**) The reflected wavelength of MIPC/NIPC under different detection times. (**B**) Changes in reflected wavelengths (Δλ) of MIPC and NIPC under loading with SMZ, SD, and SIZ solutions. (**C**) Changes in reflected wavelengths (Δλ) of MIPC sensor under several adsorption–elution cycles. (**D**) SEM image of MIPC sensor after five cycles of adsorption/elution. (**E**) Linear curve of MIPC for changes in reflected wavelengths (Δλ) and concentrations.

From Figure 6A, we can see that the diffraction wavelength of the MIPC and NIPC sensors significantly increased with time in the range of 0–3 min, but there was no significant difference from 3 to 5 min. To reduce the detection time, 3 min was used as the optimum detection time in further studies. Meanwhile, the fast response of the MIPC sensor could be attributed to the three-dimensional anti-protein structure with interconnected large pores, which reduces the diffusion resistance of molecules in the MIPC [28]. Otherwise, the change in the diffraction wavelength of the MIPC was much greater than that of the NIPC because of the existence of imprinted sites. These results show that the sensor performs with a fast response time and is suitable for rapid screening of SMZ in the field.

#### 3.4.2. Selectivity of the MIPC Sensor

The specificity of the MIPC sensor depends on whether the chemical structure of the template molecule exactly fits with the molecularly imprinted cavity. SD and SIZ, at concentrations ranging from 10^−4^ to 1 mg/L, were selected as structural analogs of SMZ to evaluate the selectivity of the MIPC sensor. From Figure 6B, we can see that the reflectance wavelength of the MIPC sensor increased from 1.5 nm to 27.7 nm when the SMZ concentration increased from 10^−4^ to 1 mg/L, which is much stronger than that of SD and SIZ (1.2–13.2 nm). There was not much difference in the reflected wavelength changes of the NIPC sensor for the three SAs, indicating that the MIPC sensor for template SMZ had specific adsorption due the existence of imprinted sites.

#### 3.4.3. Reusability of the MIPC Sensor

To confirm the reusability of the MIPC sensor, it was first immersed in blank phosphate buffer to achieve the swelling equilibrium, and the initial diffraction peak positions were detected. Next, the MIPC was immersed in 10^3^ mg/L SMZ solution to enable complete adsorption, and the diffraction peaks were measured again after reaching adsorption equilibrium. Then, the SMZ molecules were removed through the elution process to relieve the imprinted sites. The above adsorption–desorption cycle was repeated several times, and the changes in the diffraction peaks were recorded by the fiber-optic spectrometer.

From Figure 6C, we can see that there was no significant change in Δλ after five adsorption and desorption cycles. However, the excessive cycles could result in a decrease in the maximum wavelength and cause a few cracks in the imprinted framework, affecting the performance of the optical crystal and the accuracy of the detection method (see Figure 6D. This could be attributed to two reasons: (1) the damaging effect of the shaking process on the imprinted framework; or (2) a decrease in the binding sites due to the adsorption–desorption cycle. 

#### 3.4.4. Application to Real Samples

The proposed MIPC sensor for SMZ detection was then validated, with the results shown in Figure 6E. A calibration curve was established based on changes in wavelength versus the logarithms of analyte concentration (10^−4^ to 10 mg/L). A good linear relationship (Δλ = 6.25949 lg(C) + 26.52356), with a good linear correlation coefficient of 0.998, was achieved. The detection limit (LOD) was calculated to be 1.16 μg/L (3 S/N). 

To further evaluate the practical applicability of the method, chicken and milk were selected as actual samples. The results are presented in Table 1. First, no residue of SMZ was observed through the HPLC analysis. The recoveries of the milk samples and chicken samples ranged from 80.56% to 103.59%, and from 80.77% to 102.48%, respectively, with RSD < 6.41%. Compared with other reported methods shown in Table 2, our established method performed over a wider linear range. It is worth noting that the whole detection process can be completed within 3 min, which is much shorter than that of other reported methods and could meet the requirements for rapid detection. 

## 4. Conclusions

In this study, an MIPC sensor with features of specific recognition and fast response was synthesized for the detection of SMZ. During the detection process, SMZ could bind with specific sites in the MIPC through hydrogen bonding, causing a change in the lattice spacing of the MIPC. This ultimately results in a change in the reflected wavelength and produces a structural color change. Compared with conventional sensors, the MIPC sensor could convert the changes in chemical bonds into easily detectable optical signals without any additional modification of chemical groups and achieve fast and sensitive detection of target molecules by utilizing the optical properties of the sensor itself. The SMZ concentration showed a good linear relationship with the wavelength change in the range of 10^−4^~10 mg/L, and the LOD was 1.16 μg/L. The sensor has good selectivity, rapid detection, and reusability. It can realize a certain degree of visual detection, and provides an alternative for rapid on-site screening of SMZ in complex food matrices.

## Figures and Tables

**Table 1:** Recoveries of SMZ in milk samples and chicken samples.

Sample	Spiked Concentration (μg/L)	Intra-Day			Inter-Day		
		Found Concentration (μg/L)	Recovery (%)	RSD (%)	Found Concentration (μg/L)	Recovery (%)	RSD (%)
Milk	1	0.90	89.80	3.50	0.93	93.28	1.49
	10	9.61	96.13	4.45	8.80	87.86	2.73
	1 × 10^2^	88.00	88.00	4.84	80.56	80.56	4.78
	1 × 10^3^	1035.90	103.59	4.92	1016.90	101.69	4.86
Chicken	1	0.89	89.01	1.47	0.82	81.61	4.90
	10	10.25	102.48	6.41	8.08	80.77	5.55
	1 × 10^2^	91.71	91.71	4.35	88.14	88.14	3.30
	1 × 10^3^	922.00	92.20	3.77	1016.90	101.69	4.28

**Table 2:** Comparison parameters between our developed method and other reported methods.

Methods	Linear Range (ng/mL)	Detection Time (min)	LOD (ng/mL)	Recovery (%)	Reference
Fluorescent sensor	0.50–100	55	0.025	92.69–108.48	[29]
Molecularly imprinted electrochemical sensors	0.28–2.30	10	0.250	-	[30]
Lateral flow immunoassay	0.05–10	35	0.043	99.70–106.60	[31]
Electrochemical immunosensors	0.01–100	30	0.003	99.40–109	[32]
Fluorescence immunoassay	0.20–12.50	25	0.110	80.90–109.40	[33]
Chemiluminescence	1.85–21.57	65	0.920	88–91.10	[34]
Molecularly imprinted electrochemical sensors	2.5–250	5	1.900	100 ± 3	[35]
Molecularly imprinted fluorescent sensors	50–700	60	34	96.01–98.90	[36]
Molecularly imprinted photonic crystal sensor	10^−1^–10^4^	3	1.160	80.56–103.59	This work

## Data Availability

The original contributions presented in the study are included in the article; further inquiries can be directed to the corresponding author.
